# UGT2B17 Polymorphism and Risk of Prostate Cancer: A Meta-Analysis

**DOI:** 10.1155/2013/465916

**Published:** 2013-09-09

**Authors:** Marce-Amara Kpoghomou, Joella Eldie Soatiana, Fatch W. Kalembo, Ghose Bishwajit, Wei Sheng

**Affiliations:** ^1^Department of Epidemiology and Biostatistics, School of Public Health, Tong Ji Medical College, 13 Hang Kong Road, Wuhan 430030, China; ^2^Department of Ophthalmology, Tong Ji Medical College, 13 Hang Kong Road, Wuhan 430030, China; ^3^Department of Maternal and Child Health, School of Public Health, Tong Ji Medical College, 13 Hang Kong Road, Wuhan 430030, China; ^4^Department of Nutrition and Food Hygiene, School of Public Health, Tong Ji Medical College, 13 Hang Kong Road, Wuhan 430030, China

## Abstract

*Objective*. Recent studies on the association between uridine diphosphosglucuronosyltransferases (UGTs) 2B17 polymorphism and risk of prostate cancer (PCa) showed inconclusive results. To clarify this possible association, we conducted a meta-analysis of published studies. *Methods*. We searched the published literature from PubMed, Embase, Google Scholar, and China National Knowledge Infrastructure (CNKI). According to our inclusion criteria, studies that observed the association between UGT2B17 polymorphism and PCa risk were included. The principal outcome measure was the adjusted odds ratio (OR) with 95% confidence interval (CI) for the risk of PCa associated with UGT2B17 polymorphism. *Results*. A total of 6 studies with 7,029 subjects (3,839 cases and 3,190 controls) were eligible for inclusion in the meta-analysis. Overall, there was a significant association between UGT2B17 polymorphism and increased risk of prostate cancer (OR = 1.74, 95% CI 1.14–2.64, *P* < 0.001). Similar results were found in the subgroup analyses by ethnicity and types of controls. *Conclusion*. This meta-analysis demonstrates that UGT2B17 polymorphism is associated with prostate cancer susceptibility, and it contributes to the increased risk of prostate cancer.

## 1. Introduction

Prostate cancer is the fourth most common cancer in men, comprising approximately one-eighth of all male-specific cancers in the world [1]. Identifying risk factors for prostate cancer is critically important to develop potential interventions and to expand our understanding of the biology of this disease [2, 3]. The etiology of prostate cancer remains unknown, but race, age, family history of prostate cancer, and steroid hormone levels have been suggested as contributing factors [4]. The literature has revealed that eunuchs (men lacking testosterone) do not develop prostate cancer [5], generating the theory that testosterone plays a vital role in the development and progression of the disease. As a corollary, testis ablation is a well-established and effective way to stop progression of the disease [6]. Androgens, responsible for the healthy growth and maintenance of the prostate, are steroid hormones expressed in the prostate [7]. The two main types of androgens found in males, testosterone and dihydrotestosterone (DHT), have been postulated to modify the risk of prostate cancer [8, 9]. Most epidemiologic studies of prostate cancer have focused on the genes involved in the steroidogenic pathway, such as P450 cytochrome 3A4 (CYP3A4), CYP17, and SRD5A2. Although differences in androgen levels may also reflect variations in catabolism/inactivation, few studies have evaluated this hypothesis [10–13]. Different ethnic groups differ in the risk to develop prostate cancer, Afro-Americans having the highest incidence followed by Caucasians and Asians having the lowest incidence [14]. Lower levels of testosterone metabolites such as androsterone glucuronide and androstanediol glucuronide were observed in the plasma of Asian subjects [15, 16]. This finding was interpreted as a sign of lower androgen “load,” which may contribute to the lower incidence of prostate cancer in Asians. Studies indicate that environmental and lifestyle factors account for 10–15% of the racial differences in risk [17]. Uridine diphosphosglucuronosyltransferases (UGTs) constitute a family of enzymes that glucuronidate a wide variety of substrates including exogenous and endogenous compounds such as bilirubin, bile acids, and steroids. The glucuronidated product is more polar, water soluble, and more easily excreted in the bile and urine. On the basis of homology of protein primary structure, the UGT enzymes have been grouped into two families, UGT1 and UGT2 [18]. Enzymes of the UGT2 family are encoded by separate genes and are subdivided into two subfamilies, UGT2A and UGT2B. Seven functional UGT2B enzymes have been identified in humans to date, all of which are important in the homoeostasis and metabolism of steroids. UGT2B17 is one of the UGT2B enzymes and is highly expressed in the prostate [19]. It has been found to have the highest activity for androsterone, testosterone, and dihydrotestosterone [20] as compared with the other UGT2B members. Previous studies have demonstrated deletion of the *UGT2B17* gene [21, 22].

To date, a number of molecular epidemiological studies have been conducted to evaluate the effect of the UGT2B17 deletion on risk of prostate cancer [23–28]. However, until now, those studies that investigated associations between the UGT2B17 polymorphism and prostate cancer risk have yielded inconsistent results. Considering the potential important role of UGT2B17 in prostate cancer, a meta-analyses of published studies was conducted to assess the association between UGT2B17 polymorphism and cancer risk.

## 2. Materials and Methods

### 2.1. The Literature Search

Relevant studies to be included in the study were searched from the following databases PubMed, Embase, Google Scholar, and China National Knowledge Infrastructure (CNKI). The search was conducted in December, 2012. Relevant publications were identified using the following search strategies: “Uridine diphosphoglucuronosyl tranferases 2B17” or “UGT2B17” or “UGT2B17 deletion” or “polymorphism” and “prostate cancer” or “prostate carcinoma”. Additional literature was collected from cross references of both original and review articles. Only original published studies with fulltext articles were included.

### 2.2. Inclusion and Exclusion Criteria

All human associated studies were included, if they met the following criteria: (1) evaluation of the UGT2B17 present/null polymorphism and prostate cancer risk, (2) case control studies, (3) report on an OR with 95% CI, and (4) being original and published in English with an availability of a full-text. Exclusion criteria were (1) insufficient original data for the calculation of odds ratios (ORs) with corresponding 95% confidence intervals (95% CIs), (2) not case control study, and (3) review studies.

#### 2.2.1. Data Extraction

Information was carefully extracted from all the eligible publications independently by two authors according to the criteria listed previously. Disagreements were resolved by discussion among all authors. The following information was recorded for each study: first author, year of publication, country or region of origin, ethnicity, OR (95% CI), number of cases and controls, source of control group (population or hospital based), and genotype frequency of UGT2B17. For studies that included subjects of different ethnic descents, data were extracted separately for each ethnicity, which was categorized as Caucasian, Whites, and African Americans.

### 2.3. Statistical Analysis

The strength of the association between UGT2B17 polymorphism and prostate cancer risk was assessed by calculating the pooled OR with its 95% CI. The pooled ORs were obtained using either the fixed-effect (Mantel-Haenszel's method) [29] or random-effect (DerSimonian and Laird method) models [30], and the significance of the pooled OR was determined by the *Z*-test. Heterogeneity assumption was checked by the Chi-square test based *Q* statistic [31] and the *I* ^2^ statistic [32]. A significant *Q* statistic (*P* < 0.10) or *I* ^2^ statistic (*I* ^2^ > 50%) indicated obvious heterogeneity across studies, and the random-effect model was selected to pool the ORs. Otherwise, the fixed-effect model was selected to pool the ORs. Subgroup analyses were performed by ethnicity and types of controls. Subgroup analyses were firstly performed by ethnicity, and ethnicities were categorized as Caucasians, Whites, and African Americans. Finally, subgroup analyses were performed by the types of controls. Sensitivity analysis was performed to assess the stability of results. Publication bias was investigated with the funnel plot. The funnel plot should be asymmetric when there is a publication bias, and the funnel plot asymmetry was further assessed by the method of Egger's linear regression test [33]. All statistical analyses were performed using STATA statistical software (version 10.1; Stata Corporation, College Station, USA).

## 3. Results

### 3.1. Eligible Studies

**Figure 1:**
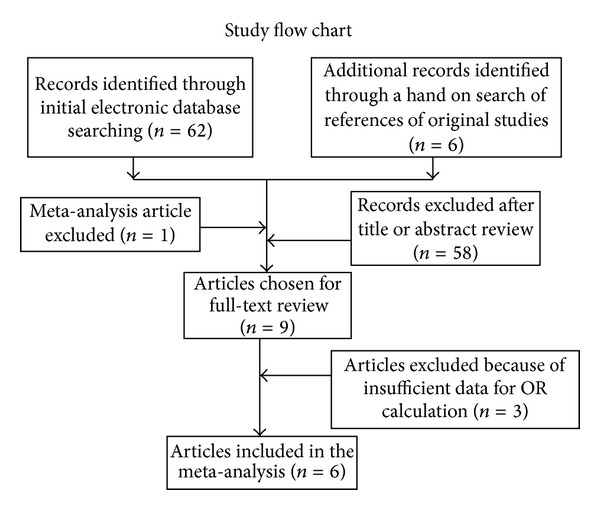
The flow chart of included studies in the meta-analysis.

The flow chart of study selection for this meta-analysis is presented in Figure 1. A total of 69 published records up to December 15, 2012, were identified, of which 9 were considered potentially eligible for inclusion in this meta-analysis and were retrieved in full texts. Among these, 3 studies were excluded because they had insufficient data on OR calculation [34–36]. Finally, 6 articles were included in the meta-analysis. In addition, the study investigating multiple ethnicities [28] was separated into two studies in the subgroup analysis.

The main characteristics of the included studies are presented in Table 1. Overall, 6 publications, including 3,839 cases and 3,190 controls, were included in this meta-analysis. Among the 6 studies included in this meta-analysis, 4 studies were conducted on Caucasians, 1 on Whites, and 1 on African Americans and Caucasians. 

### 3.2. Meta-Analysis

**Figure 2:**
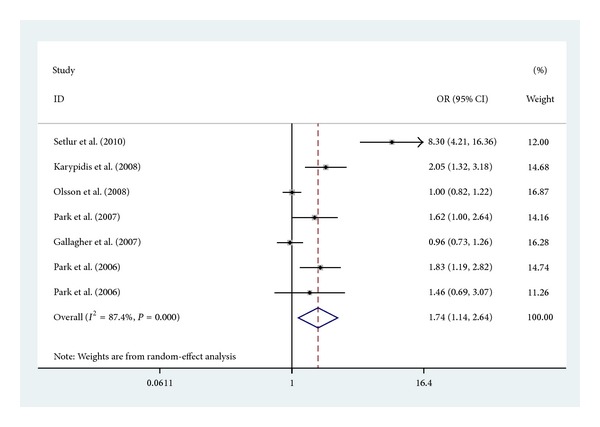
Forest plot (random-effect model) of meta-analysis regarding association between UGT2B17 polymorphism and prostate cancer risk. The squares and horizontal lines correspond to the study-specific OR and 95% CI. The area of the squares reflects the weight (inverse of the variance). The diamonds represent the summary OR and 95% CI.

The summary of meta-analysis for UGT2B17 polymorphism with prostate cancer risk is shown in Table 1. Overall, there was a significant association between UGT2B17 polymorphism and increased risk of prostate cancer (OR = 1.74, 95% CI 1.14–2.64, *P* < 0.001) (Figure 2). 

**Figure 3:**
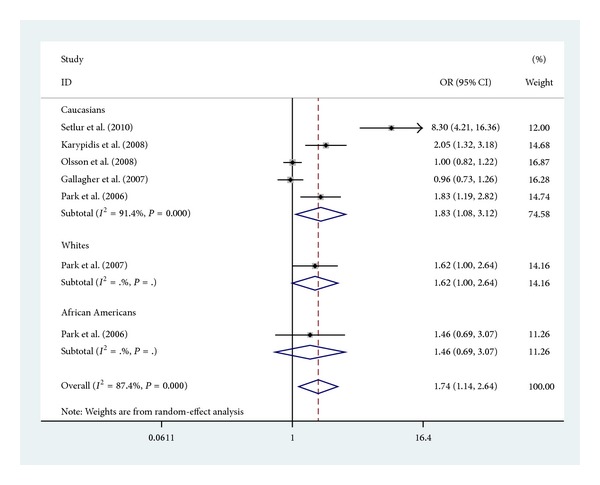
Forest plot (random-effect model) of meta-analysis regarding association between UGT2B17 polymorphism and ethnicity. The squares and horizontal lines correspond to the study-specific OR and 95% CI. The area of the squares reflects the weight (inverse of the variance). The diamonds represent the summary OR and 95% CI.

**Figure 4:**
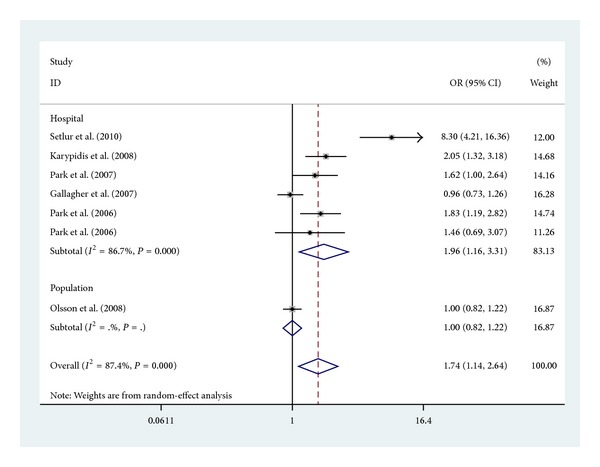
Forest plot (random-effect model) of meta-analysis regarding association between UGT2B17 polymorphism and sources of controls. The squares and horizontal lines correspond to the study-specific OR and 95% CI. The area of the squares reflects the weight (inverse of the variance). The diamonds represent the summary OR and 95% CI.

Subgroup analyses were firstly performed by ethnicity (Caucasians, whites, African Americans). There was an obvious association between UGT2B17 polymorphism and increased risk of prostate cancer in Caucasians (OR = 1.83, 95% CI 1.08–3.12, *P* = 0.026) (Figure 3). In the subgroup analysis which were performed by types of controls (population and hospital based), there was an obvious association between UGT2B17 polymorphism in hospital based and increased risk of prostate cancer (OR = 1.96, 95% CI 1.16–3.31, *P* = 0.011) (Figure 4).

### 3.3. Evaluation of Heterogeneity

To examine the effect of heterogeneity between studies included in the meta-analysis results, subgroup analyses stratified by the following factors were conducted: ethnicity and sources of controls. The results were as follows: ethnicity (*P* = 0.026 and *I* ^2^ = 91.4% for Caucasians) and sources of controls (*P* = 0.011 and *I* ^2^ = 86.7% for hospital based). UGT2B17 polymorphism was significantly associated with prostate cancer risk in overall analysis (*I* ^2^ = 87.4%).

### 3.4. Sensitivity Analysis

**Figure 5:**
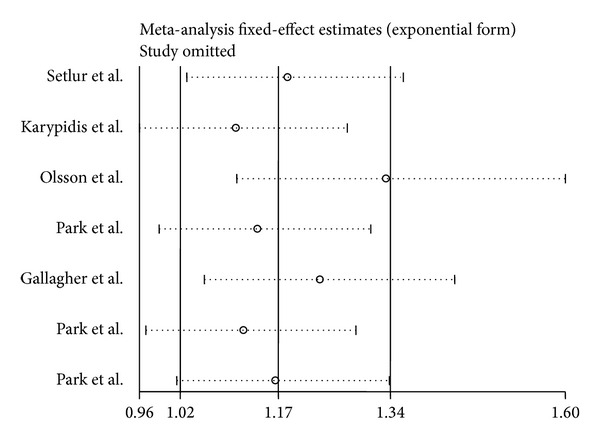
Sensitivity analysis of the summary odds ratio coefficients on the association between UGT2B17 polymorphism and prostate cancer risk. Results were computed by omitting each study in turn. Meta-analysis random-effect estimates (exponential form) were used. The two ends of the dotted lines represent the 95% CI.

In the sensitivity analysis, the influence of each study on the pooled OR was examined by repeating the meta-analysis while omitting each study, one at a time. This procedure confirmed the stability of our overall result (Figure 5). 

### 3.5. Publication Bias

**Figure 6:**
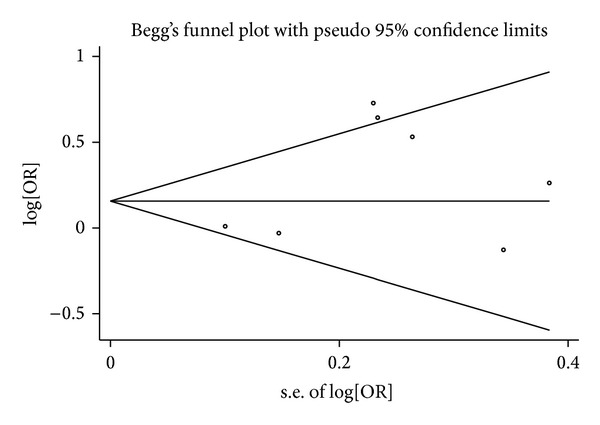
Begg's funnel plot for publication bias test (for Null versus Present). Each point represents a separate study for the indicated association. Log[OR]: natural logarithm of OR. Horizontal line of mean effect size.

Begg's funnel plot and Egger's test were conducted to assess a possible publication bias in the literature. The shapes of funnel plots did not reveal any evidence of funnel plot asymmetry. The results Egger's test showed no indication of publication bias (*P* = 0.19; Figure 6). 

## 4. Discussion

Genetic susceptibility to cancer has been a research focus and many genetic association meta-analyses have been published to find some possible susceptibility polymorphisms [3]. Previous study assessing the association between UGT2B17 deletion and the risk of prostate cancer reported inconclusive and inconsistent findings. Therefore, to get a reliable conclusion of the association between UGT2B17 polymorphism and prostate risk, we conducted the present meta-analysis of 6 independent studies including a total of 7,029 subjects (3,839 cases and 3,190 controls) [23–28]. Overall, there was a significant association between UGT2B17 status and increased risk of prostate cancer (Figure 2). Moreover, the association remained significant among Caucasians and hospital-based subgroup analysis. 

The UGT2B17 enzyme is particularly active in androgen glucuronidation and is highly expressed in prostate tissue [19, 20, 37]. The data are consistent with the hypothesis that the UGT2B17 enzyme may play a role in degradation of dihydrotestosterone [38] and that an excessive amount of dihydrotestosterone may be associated with carcinogenesis in the prostate tissue [39]. Unfortunately, most of studies have only a few hundred participants, even less, which is too small to precisely evaluate the overall effects. Meta-analysis has been considered to be a powerful tool to overcome this problem by combining the results from independent studies together. This study has shown that the UGT2B17 polymorphism may be involved in the development of prostate cancer. A previous meta-analysis indicated that there was a marginally significant association with the UGT2B17 Del polymorphism under Del/Del versus Ins/Ins +Ins/Del (*P* = 0.05) [40]. It is known that the allele frequencies of metabolic genes are not equally distributed throughout the human population but follow diverse ethnic patterns; therefore, the subgroups according to ethnicity were performed. Our results indicated that significant prostate cancer risks of people with UGT2B17 polymorphism are in Caucasians with OR = 1.83 (1.08–3.12) and *P* = 0.026. Ethnicity is a well-established confounding factor for prostate cancer risk. It was previously reported that the UGT2B17 deletion polymorphism was not associated with an increased risk of prostate cancer in African-Americans from Arkansas [13] but was associated with an increased risk (OR = 1.9) in Caucasian subjects from Florida that included 293 cases and a similar number of controls [13]. Obviously, the genetic variation in UGT2B17 (and UGT2B15) does not explain the ethnic differences observed in prostate cancer that was discussed previously [41]. The interethnic differences observed for the UGT2B17 polymorphism frequency are consistent with the ethnic distribution of the UGT2B15 Y85D polymorphism. On one hand, it is conceivable that an increased local enzymatic degradation in the prostate may decrease the androgen exposure of the androgen receptors thereby counteracting the postulated proliferative role of these receptors. On the other hand, UGT2B17 and *UGT2B15* alleles that are associated with an increased risk of prostate cancer are more common in Asian populations than in Caucasian populations. Obviously, the genetic variation in UGT2B17 (and UGT2B15) does not explain the ethnic differences observed in prostate cancer that was discussed previously [41]. Additionally, as limited sample size may not have enough statistical power to detect a real effect or generate a fluctuated estimation, the small sample size of whites and African Americans in this meta-analysis should also be taken into consideration.

Furthermore, the results of this meta-analysis showed that UGT2B17 polymorphism has strikingly increased the risk of prostate cancer risk susceptibility when stratified by control source. However, we obtained the highest risk of prostate cancer in hospital-based controls with OR = 1.96 (1.16–3.31) and *P* < 0.001. The possible reason may be that UGT2B17 polymorphism could influence the susceptibility to noncancer diseases, so its genotype frequency possibly differed between the hospital-based and population-based controls. Also the small sample size of population based in this meta-analysis should be taken into consideration.

If significant heterogeneity is present, pooled summary estimates from such meta-analyses are hard to interpret. In our meta-analysis, obvious heterogeneity across studies was observed in the overall comparison and some group analyses.

The current meta-analysis has vital advantages compared to other studies; however, there are some limitations in this meta-analysis. Firstly, only published studies in English with full text were included in this meta-analysis; therefore, the publication bias may have occurred. Secondly, the small sample size in subgroup analyses may have limited statistical power. Furthermore, due to limited studies included in this meta-analysis, we were unable to perform further subgroup analyses such as by genotyping method. Thirdly, the results of subgroup analysis should be interpreted with caution because of the limited statistical power. We anticipate that issues will be addressed in future studies. Finally, most studies in the meta-analysis were retrospective design which could suffer more risk of bias owing to the methodological deficiency of retrospective studies. Though there was no obvious risk of publication bias in the present meta-analysis, the risks of other potential bias were unable to be excluded. Therefore, more studies with prospective design and low risk of other bias are needed to provide a more precise estimate of the association between UGT2B17 polymorphism and prostate cancer.

In summary, this meta-analysis with a total of 3,839 cases and 3,190 controls suggests that the UGT2B17 polymorphism is associated with increased risk of prostate cancer in Caucasians; well-designed studies with large sample sizes involving various ethnic populations are warranted.

## Figures and Tables

**Table 1:** Characteristics of studies included in the meta-analysis.

First author	Year	Country	Ethnicity	Samples size (case/control)	OR (95% CI)	Source of controls	Genotyping method^c^
Setlur [23]	2010	Austria	Caucasians	121/205	0.88 (0.45–1.73)^a^	Hospital based	PCR
Karypidis [24]	2008	Sweden	Caucasians	174/161	2.07 (1.32–3.25)^a^	Hospital based	RT PCR
Olsson et al. [25]	2008	Sweden	Caucasians	2,480/1,672	1.01 (0.83–1.23)^b^	Population based	Multiplex PCR
Park [26]	2007	USA	Whites	247/273	1.7 (1.03–2.9)^a^	Hospital based	PCR-RFLP
Gallagher [27]	2007	USA	Caucasians	411/397	0.97 (0.73–1.30)^a^	Hospital based	RT PCR
Park [28]	2006	USA	Caucasian	293/367	1.9 (1.2–3.0)^a^	Hospital based	RT PCR
			African Americans	113/115	1.3 (0.6–2.7)^a^		

^a^Adjusted odds ratio (OR) and 95% confidence interval (CI).

^b^Unadjusted odds ratio (OR) and 95% confidence interval (CI).

^c^Genotyping method—PCR: polymerase chain reaction, RT-PCR: real-time polymerase chain reaction, and PCR-RFLP: polymerase chain reaction-restriction fragment length polymorphism.
